# Barreras al acceso de los servicios de salud a las personas con enfermedades huérfanas o raras en el departamento del Huila, Colombia

**DOI:** 10.7705/biomedica.7991

**Published:** 2026-06-01

**Authors:** Ingrid Yolercy Troche, William González, Anyi Daniela Montealegre, Brayant Andrade

**Affiliations:** 1 Departamento de Enfermería, Universidad Surcolombiana, Neiva, Colombia Universidad Surcolombiana Universidad Surcolombiana Neiva Colombia; 2 Secretaría de Salud Departamental del Huila, Neiva, Colombia Secretaría de Salud Departamental del Huila Neiva Colombia

**Keywords:** enfermedades raras, accesibilidad a los servicios de salud, barreras de acceso a los servicios de salud, política de salud, factores socioeconómicos, inequidades en salud, Rare diseases, health services accessibility, barriers to access of health services, health policy, socioeconomic factors, health inequities

## Abstract

**Introducción.:**

A pesar de la obligatoriedad de brindar atención prioritaria y oportuna a las personas con enfermedades huérfanas o raras, esta población continúa encontrando barreras al acceso de los servicios de salud.

**Objetivo.:**

Identificar las barreras al acceso de los servicios de salud para las personas con diagnóstico de enfermedades huérfanas o raras en el departamento del Huila, 2022.

**Materiales y métodos.:**

Se trata de un estudio transversal analítico a partir de datos secundarios de 134 personas con diagnóstico de una enfermedad huérfana o rara en el departamento del Huila. Se hizo caracterización sociodemográfica y clínica; además, se identificaron las barreras para el acceso a los servicios de salud a nivel geográfico, económico, social, de conocimiento y cultura, y aquellas propias de la atención en salud. El análisis estadístico con medidas de tendencia central y de asociación en Stata 15™.

**Resultados.:**

La mediana de edad fue de 15 años, el 51,5 % era de sexo masculino y el 61,9 % vivía en el área urbana y de ingresos económicos bajos. El diagnóstico más prevalente fue el de síndrome de Guillain-Barré y el 32,8 % requirió hospitalización por complicaciones de la enfermedad.

El vivir en el área rural implicó mayores probabilidades de presentar limitaciones para llegar al centro de atención (OR = 10,7; IC_95%_: 4,3-27) y escasez de dinero para transportarse (OR = 2,84; IC_95%_: 1,26-6,52). Hubo asociación estadística entre el régimen subsidiado y la falta de entrega de los medicamentos (OR = 4,59; IC_95%_: 1,44-19,17).

**Conclusión.:**

Los participantes enfrentan barreras significativas de acceso a los servicios de salud asociados con vivir en el área rural y el régimen de afiliación.

## Abstract (en)

**Introduction.:**

Despite the obligation to provide priority and timely care to people with orphan diseases, they continue to find barriers to health services.

**Objective.:**

To determine access to health services barriers for people diagnosed with orphan or rare diseases.

**Materials and methods.:**

We carried out a cross-sectional analytical study based on secondary data of 134 people reported with orphan or rare diseases in the department of Huila. Sociodemographic and clinical characterization and identification of barriers to access to health services at the geographic, economic, social, knowledge, cultural and health care levels were carried out. Statistical analysis was performed in Stata 15™ using measures of central tendency and association.

**Results.:**

The median age was 15 years, 51.5% were male, 61.9% lived in urban areas, low income predominated, the most prevalent diagnosis was Guillain-Barré syndrome and 32.8% required hospitalization for complications of the disease. Living in rural areas presents a greater probability of having limited access to the center of care (OR = 10.7; 95% CI: 4.3-27) and lack of money for transportation (OR = 2.84; 95% CI: 1.26-6.52). Statistical association between subsidized regime and non-delivery of medicines (OR = 4.59; 95% CI: 1.44-19.17) was found.

**Conclusion.:**

Participants face significant barriers to accessing health services associated with living in rural areas and their affiliation status.

En Colombia, las enfermedades huérfanas hacen parte de las enfermedades raras, las ultrahuérfanas y las olvidadas, caracterizadas por su heterogeneidad, complejidad y diversidad. Además, por ser en su mayoría debilitantes, degenerativas o progresivas, son una amenaza para la vida ^1^^-^^3^ y por tener una prevalencia menor de 1 por 5000 personas ^4^. Estas enfermedades agrupan, aproximadamente, 6000 enfermedades raras en el mundo ^5^.

En Colombia, mediante la Resolución 023 de 2023 del Ministerio de Salud y Protección Social ^6^, se reconocen 2247 enfermedades de este tipo, con una afectación del 6 al 8 % de la población, y constituyen un desafío para la salud pública ^5^.

Las enfermedades huérfanas o raras, al tener manifestaciones clínicas complejas e inespecíficas, presentan demoras para recibir diagnóstico, tratamiento y rehabilitación oportunos, lo que puede conducir a un deterioro progresivo, con complicaciones tempranas e impacto negativo en la calidad de vida del paciente, familia y cuidadores o, en el peor de los casos, producir una muerte prematura ^2^. La atención en salud que reciben estos pacientes es fragmentada, poco eficiente y carente de atención biopsicosocial y multidisciplinar ^1^.

Teniendo en cuenta la importancia que representan los casos de enfermedades huérfanas o raras en el sistema de salud colombiano, se normatizó la implementación de un sistema de información de estos pacientes mediante la Ley 1392 de 2010 ^7^. Por lo anterior, el Instituto Nacional de Salud estableció la notificación obligatoria al Sistema de Vigilancia en Salud Pública (SIVIGILA) en el evento 342 ^8^. No obstante, existe un subregistro de casos debido a la falta de conocimiento técnico-científico, la deshumanización del personal médico, la escasa experiencia de su manejo de los prestadores primarios y al alto costo de los tratamientos para este tipo de enfermedades.

Además, los afiliados al Sistema General de Seguridad Social en Salud tienen derecho a todas las tecnologías y servicios disponibles y autorizados en el país por la autoridad competente; sin embargo, solo el 31,4 % de las enfermedades huérfanas o raras están incluidas en el plan de beneficios, desfavoreciendo la salud y el bienestar del porcentaje restante de los pacientes diagnosticados ^3^.

Hay evidencia de las barreras de acceso que enfrentan los pacientes con enfermedades huérfanas o raras y sus familias en el territorio colombiano, que confluyen en atención inoportuna, ineficaz y de baja calidad ^4^. A pesar de que la ley antitrámites ^9^ y la Ley Estatutaria 1751 de 2015 ^10^ han regulado los procesos administrativos, persiste el incremento de los tiempos de respuesta para los trámites de autorización, dispensación y asignación de las citas médicas especializadas, lo que influye negativamente en la adhesión terapéutica de los pacientes ^4^.

En este estudio se entienden como «barreras de acceso en salud» a los obstáculos o dificultades que tiene una persona o un grupo de personas para buscar y obtener atención en salud cuando y donde sea necesario. El concepto de «acceso» presenta una connotación multidimensional al incluir características de disponibilidad, accesibilidad, acomodación y aceptabilidad ^11^^-^^13^.

Ante el panorama planteado, y ante la ausencia de investigaciones sobre el tema en el departamento del Huila, era necesario determinar las barreras al acceso de los servicios de salud en personas diagnosticadas con enfermedades huérfanas o raras en este departamento del sur de Colombia en el 2022.

## Materiales y métodos

Se llevó a cabo un estudio transversal analítico con fuentes de información secundarias, reportadas a una base de datos de los pacientes con enfermedades huérfanas o raras. La base de datos fue facilitada por la Secretaría de Salud Departamental del Huila, sin los datos de identificación personal, según la política de tratamiento de datos personales de la Ley 1581 del 2012 ^14^.

Se incluyeron todos los casos notificados hasta el año 2022 en la ficha de notificación individual de los datos complementarios para las enfermedades huérfanas o raras, código INS 342. Se excluyeron los casos que tenían información incompleta, que informaban un municipio de residencia por fuera del departamento del Huila y las fichas duplicadas.

La ficha fue diligenciada por un profesional de la salud, incluyendo información de datos sociodemográficos y diagnóstico según la Clasificación Internacional de Enfermedades 10 (CIE-10). Además, la ficha contaba con un anexo para ampliar la información sobre la percepción de las relaciones familiares, la disponibilidad de recursos económicos y el acceso a los servicios de salud en torno al diagnóstico y tratamiento de la enfermedad de base.

Las variables analizadas fueron las siguientes.

### Sociodemográficas

1. Sexo como condición biológica.
2. Edad según el curso de vida: primera infancia (0 a 5 años), infancia (6 a 11 años), adolescencia (12 a 18 años), juventud (14 a 26 años), adultez (27 a 59 años) O vejez (60 años y más) ^15^.
3. Etnias reconocidas en Colombia.
4. Área de residencia, en urbana o rural.
5. Ubicación del municipio donde residía; se agruparon los 37 municipios en cuatro subregiones (centro, norte, occidente y sur), siguiendo la organización usada por los documentos oficiales de la Secretaría de Salud Departamental del Huila.
6. Régimen de afiliación en salud: régimen subsidiado (personas pobres y vulnerables del país, sin capacidad de pago) y régimen con capacidad de pago que incluyó a las personas afiliadas al régimen contributivo y regímenes especiales o de excepción (fuerzas militares, Ecopetrol y profesores del magisterio). Es importante aclarar que, en Colombia, la afiliación en salud se efectúa en los regímenes mencionados mediante las aseguradoras o entidades promotoras de salud, que son las entidades responsables de la afiliación y prestación del plan de beneficios a sus usuarios ^16^.
7. La estructura familiar se categorizó según las relaciones, el número y ciclo de vida de los integrantes que conviven en la vivienda: familia nuclear, monoparental, reconstituida y extensa ^17^.
8. Los ingresos mensuales se reportaron en los términos del salario mínimo legal mensual vigente en Colombia para el año 2022, por valor de COP$ 1 000 000.

Clínicas

1. Diagnóstico médico principal, el cual estaba confirmado como una enfermedad huérfana o rara, según la CIE-10.
2. Asimismo, reportaron si presentaba algún tipo de discapacidad, las especialidades médicas a las cuales asistía para el diagnóstico y el tratamiento que recibía.

Las barreras de acceso a los servicios de salud se agruparon en cinco categorías:

1. ***Geográficas:*** integradas por cinco ítems relacionados con el tiempo, el medio de transporte y la accesibilidad a las instituciones prestadoras de salud (entendiéndose dentro de estas los centros de salud, clínicas, hospitales y consultorios médicos).
2. ***Económicas:*** con cinco ítems, identificando la disponibilidad de dinero para acceder a los medios de diagnóstico y los tratamientos de su enfermedad de base.
3. ***Sociales:*** con tres ítems sobre la relación con el contexto.
4. ***De conocimiento y cultura:*** con tres ítems relacionados con información recibida de los profesionales de la salud sobre el diagnóstico y el tratamiento.
5. ***Prestación de los servicios de salud:*** sobre la efectividad y la oportunidad del asegurador en gestionar los trámites administrativos para la prestación de los servicios de salud.

Se hizo un análisis estadístico descriptivo para las variables cualitativas con sus frecuencias absolutas y relativas. Para las variables cuantitativas, se aplicaron medidas de tendencia central (media, mediana) con previa verificación de la normalidad por medio de la prueba de Kolmogorov-Smirnov.

Posteriormente, se llevó a cabo un análisis inferencial con enfoque bivariado. Para comparar los promedios entre dos grupos, se utilizó la prueba de t de Student o la prueba de U de Mann-Whitney dependiendo del cumplimiento de la normalidad. Para determinar la asociación entre las barreras de accesibilidad a los servicios de salud según el área de residencia y el régimen de afiliación en salud, se usó el OR *(odds ratio)* con sus respectivos intervalos de confianza (IC_95%_), considerando una p menor de 0,05 como estadísticamente significativa. El análisis se realizó en el *software* Stata 15™.

La presente investigación se consideró con un riesgo menor mínimo, según la Resolución 8430 de 1993 del Ministerio de Salud de Colombia, que normatiza la investigación en seres humanos ^18^, dado que el análisis de los datos se realizó a partir de una fuente de información secundaria, obtenida en el proceso de notificación individual en la ficha epidemiológica 342 del SIVIGILA y su anexo de información ampliada.

Asimismo, para garantizar los principios bioéticos de la confidencialidad de los pacientes, la base de datos aportada no contaba con datos de identificación de los participantes (nombres, apellidos, números de identificación, dirección ni teléfono).

## Resultados

### Caracterización sociodemográfica

Participaron 134 personas con enfermedades huérfanas o raras, que cumplieron con los criterios de selección. La mediana de la edad fue de 15 años (rango intercuartílico, RIQ = 36); edad mínima de 1 y máxima de 82 años. El 52,9 % de los pacientes eran menores de edad y el 51,5 % de sexo masculino.

Para el momento del estudio, 6 de cada 10 personas vivían en el área urbana. El 66,4 % (89/134) estaba ubicado en las regiones norte y centro del departamento y el 17,2 % (23/134) vivía en Neiva, la capital del departamento. El 68,7 % (92/134) estaba afiliado al régimen subsidiado de salud y la séptima parte eran beneficiarios de dos aseguradoras (38,8 % (52/134), Nueva EPS, y el 35,1 % (47/134), Sanitas).

En cuanto al nivel académico, el 53 % (71/134) de la población manifestó contar con el grado de escolaridad básica primaria y secundaria; el 15,7 % (21/134) indicó contar con educación superior y el 31,3 % (42/134) no reportó este dato. Cabe resaltar que, a nivel rural, la escolaridad fue más baja que a nivel urbano, dado que, en el primer grupo, el 43,1 % (22/51) reportó no tener ningún nivel de estudios y el 33,4 % (17/51), básica primaria; por el contrario, a nivel urbano, el 24,1 % (20/83) reportó educación superior (técnico y universitario). El 90 % (123/134) pertenecía a estratos socioeconómicos bajos, el 35,8 % (48/134) indicó que se encontraba estudiando y el 17,2 % (23/134) laborando.

A nivel del entorno familiar, 8 de cada 10 participantes refirieron pertenecer a una familia nuclear con un promedio de ingresos en el hogar inferior a un salario mínimo. El 61,9 % (83/134) manifestó que el diagnóstico de enfermedad huérfana o rara había impactado la dinámica familiar, afectando principalmente las relaciones intrafamiliares (32,8 %; 44/134) y la responsabilidad económica en el hogar. En su mayoría (67,1 %; 90/134), está a cargo de un familiar, destacándose el apoyo de padres con el mayor porcentaje. A su vez, solo el 18,7 % (25/134) de los pacientes recibía apoyos o beneficios de programas gubernamentales (cuadro 1).

**Cuadro 1:** Caracterización sociodemográfica de personas con enfermedades huérfanas (N = 134)

Variables	Categoría	n (%)
Curso de vida	Primera infancia	32 (23,9)
	Infancia	25 (18,6)
	Adolescencia	14 (10,4)
	Juventud	12 (9)
	Adultez	32 (23,9)
	Vejez	19 (14,2)
Sexo	Masculino	69 (51,5)
	Femenino	65 (48,5)
Etnia	Mestizo	128 (95,5)
	Afrocolombiano	5 (3,7)
	Indígena	1 (0,8)
Área de residencia	Urbano	83 (61,9)
	Rural	51 (38,1)
Subregiones del Huila	Norte	54 (40,3)
	Centro	35 (26,1)
	Sur	30 (22,4)
	Occidente	15 (11,2)
Régimen de salud	Subsidiado	92 (68,7)
	Contributivo	39 (29,1)
	Especial	3 (2,2)
Grado de escolaridad	Ninguno	42 (31,3)
	Primaria	39 (29,1)
	Secundaria	32 (23,9)
	Técnico	9 (6,7)
	Universitario	12 (9)
Estado civil	Casado	26 (19,4)
	Unión libre	16 (11,9)
	Soltero	90 (67,2)
	Separado	2 (1,5)
Estrato socioeconómico	I	90 (67,2)
	II	33 (24,6)
	III	7 (5,2)
	IV	2 (1,5)
	V	2 (1,5)
Ocupación	No aplica	26 (19,4)
	Estudiante	48 (35,8)
	Hogar	28 (20,9)
	Pensionado	4 (3)
	Empleado	23 (17,2)
	Desempleado	5 (3,7)
Estructura familiar	Nuclear	108 (80,6)
	Monoparental	12 (9)
	Reconstituida	7 (5,2)
	Extensa	7 (5,2)
Impacto entorno familiar	Ninguno	51 (38,1)
	Déficit de relaciones intrafamiliares	44 (32,8)
	Alteraciones psicológicas	16 (12)
	Dificultad para el apoyo de tareas del hogar	11 (8,2)
	Alteraciones económicas	11 (8,2)
	Dificultad para asignar un cuidador	1 (0,7)
Ingresos mensuales del hogar (SMMLV)	< 1	108 (80,6)
	Entre 1 y 2	22 (16,4)
	> 2	4 (3)
Carga económica	Padres	84 (62,7)
	Amigos o conocidos	44 (32,9)
	Otro familiar	5 (3,7)
	Hermanos	1 (0,7)
Apoyo gubernamental	Ninguno	109 (81,3)
	Familias en acción	15 (11,2)
	Ingreso solidario	5 (3,7)
	Tercera edad	2 (1,5)
	Otros	3 (2,3)

SMMLV: salario mínimo mensual legal vigente

### Caracterización clínica

Se reportaron 63 enfermedades huérfanas o raras, siendo el síndrome de Guillain-Barré la de mayor prevalencia (10,4 %; 14/134), seguido de la esclerosis múltiple (8,2 %; 11/134), las cuales se agruparon según la clasificación patológica de la CIE-10, destacándose, en orden descendente: las malformaciones congénitas, las deformidades y anomalías cromosómicas (26,9 %; 36/134) y las enfermedades del sistema nervioso (26,1 %; 35/134).

El 32,8 % (44/134) había requerido atención hospitalaria en algún momento de la vida para la atención de complicaciones de su enfermedad. Todos los pacientes han requerido el manejo por médico especialista, el 33,6 % (45/134) el de uno, el 53 % (71/134) de dos y el 13,4 % (18/134) de tres especialistas. Se reportaron más de veinte especialidades médicas diferentes; la más consultada fue genética (50 %; 67/134), seguida por neurología (35,8 %; 48/134). El 85,1 % (114/134) presentó algún tipo de discapacidad como producto de la enfermedad, con mayor prevalencia a nivel físico (38,1 %; 51/134) y discapacidad múltiple en el 21,6 % (29/134).

Los pacientes que pertenecen al grupo etario de la primera infancia reportaron un 96,9 % (31/32) de algún grado de discapacidad, seguido del grupo de infancia con el 88 % (22/25). Las personas que presentaron alguna discapacidad, comparadas con quienes no la presentaron, tienen más probabilidad de haber requerido hospitalización en algún momento de la vida con el fin de recibir tratamiento de las complicaciones de su enfermedad (OR = 5,25; IC_95%:_ 1,51-48,4).

Solo el 21,6 % (29/134) de los participantes recibió apoyo de rehabilitación y el 10,4 % (14/134) tiene certificado de discapacidad, y manifestó que los principales obstáculos para obtener el certificado fueron la falta de orientación del personal y de la institución de salud y los problemas administrativos de las aseguradoras y demás organismos encargados del proceso. Finalmente, el 26,9 % (36/134) de personas había necesitado hacer uso del recurso legal de tutela para exigir la prestación de un servicio de salud (cuadro 2).

**Cuadro 2:** Caracterización clínico-terapéutico de personas con enfermedades huérfanas (N = 134)

Variables	Categoría	n (%)
Diagnóstico médico	Síndrome de Guillain-Barré	14 (10,4)
	Esclerosis múltiple	11 (8,2)
	Microtia	7 (5,2)
	Gastrosquisis	6 (4,5)
	Fibrosis quística	5 (3,7)
	Artritis juvenil idiopática	5 (3,7)
	Déficit hereditario del factor VIII	4 (3)
Clasificación CIE 10	Malformaciones congénitas, deformidades y anomalías cromosómicas	36 (26,9)
	Enfermedades del sistema nervioso	35 (26,1)
	Enfermedades endocrinas, nutricionales y metabólicas	17 (12,7)
	Enfermedades del sistema osteomuscular y del tejido conjuntivo	17 (12,7)
	Enfermedades de la sangre y de los órganos hematopoyéticos y otros	17 (12,7)
	trastornos que afectan el mecanismo de la inmunidad	
	Enfermedades del aparato digestivo	3 (2,2)
Antecedentes de hospitalización	Sí	44 (32,8)
	No	90 (67,2)
Especialidad tratante	Genética	67 (50)
	Neurología	48 (35,8)
	Medicina interna	18 (13,4)
	Endocrinología	12 (9)
	Hematología	12 (9)
	Reumatología	10 (7,5)
Tipo de discapacidad	Ninguna	20 (14,9)
	Física	51 (38,1)
	Visual	11 (8,2)
	Psicosocial (mental)	10 (7,5)
	Auditiva	8 (6)
	Intelectual	5 (3,7)
	Múltiple	29 (21,6)
Apoyo terapéutico	Ninguno	105 (78,4)
	Rehabilitación física	17 (12,7)
	Psicología	7 (5,2)
	Terapia ocupacional	3 (2,2)
	Terapia neurosensorial	1 (0,7)
	Fonoaudiología	1 (0,7)
Certificado de discapacidad	No aplica	20 (14,9)
	Sí	14 (10,4)
	No	100 (74,6)
Barreras para el certificado	No requiere	20 (14,9)
	Falta de orientación	91 (67,9)
	Problemas administrativos	23 (17,2)
Tutelas por servicios de salud	Sí	36 (26,9)
	No	98 (73,1)

### Barreras para el acceso a los servicios de salud

A nivel geográfico, aproximadamente la mitad (47,7 %; 64/134) de los participantes manifestaron que se demoran más de una hora para asistir a la consulta especializada; el 73,9 % (99/134) no contaba con transporte propio para asistir a los centros de atención en salud y, entre estos, el medio de transporte más utilizado fue el bus (48,5 %; 48/99), seguido del transporte informal de mototaxi en el 20,2 % (20/99) y el 31,3 % (31/99) se desplazaban caminando o en taxi.

Asimismo, el 35,1 % (47/134) refirió problemas en las vías de acceso a los centros de atención y el 44 % (59/134) necesitaron trasladarse a otros departamentos para practicarse pruebas de diagnóstico o recibir tratamientos no disponibles en el Huila.

En cuanto a las barreras de tipo económico, los participantes refirieron que en promedio gastan COP$ 175 000 para asistir a una cita con un médico especialista o para practicarse un examen de diagnóstico. El gasto promedio de los pacientes del área rural fue de COP$ 120 050 más alto que los residentes del área urbana, con presencia de significancia estadística (p < 0,001). Más de la mitad de los pacientes (57,5 %; 77/134) manifestaron tener dificultades o no contar con el dinero suficiente para transportarse a la institución prestadora de servicios de salud.

El 30,6 % (41/134) ha tenido que pagar cuotas moderadoras o copagos para recibir atención a su enfermedad; un poco más de la tercera parte (37,3 %; 50/134) ha tenido que incurrir en gastos económicos por concepto de medicamentos o exámenes diagnósticos no autorizados por la aseguradora y el 40,3 % (54/134) ha incurrido en gastos adicionales o complementarios para acceder al tratamiento de la enfermedad, lo que sugiere la necesidad de apoyo económico para viáticos (transporte, hospedaje, alimentación), entre otros (cuadro 3).

**Cuadro 3:** Barreras de tipo geográfico y económico de personas con enfermedades huérfanas (N = 134)

Variables	Categoría	n (%)
Tiempo promedio para asistir a la IPS	Menor de 30 minutos	38 (28,4)
	Entre 30 a 60 minutos	32 (23,9)
	Entre 1 y 2 horas	44 (32,8)
	Mayor de 2 horas	20 (14,9)
Transporte propio para asistir a la IPS	Sí	35 (26,1)
	No	99 (73,9)
Medio de transporte	Propio	35 (26,1)
	Bus	48 (35,8)
	Mototaxi	20 (14,9)
	A pie	19 (14,2)
	Taxi	12 (9)
Limitaciones para el acceso a vías hacia a IPS	Sí	47 (35,1)
	No	87 (64,9)
Desplazamiento a otro departamento para la realización de exámenes o tratamiento	Sí	59 (44)
	No	75 (56)
Disponibilidad de dinero para transporte hacia la IPS	Sí	57 (42,5)
	No	77 (57,5)
Pago de cuotas moderadoras o copagos	Sí	41 (30,6)
	No	93 (69,4)
Pago por medicamentos o por exámenes diagnósticos	Sí	50 (37,3)
	No	84 (62,7)
Gastos adicionales para llevar a cabo el tratamiento	Ninguno	80 (59,7)
	Viáticos	44 (32,8)
	Tratamientos no incluidos en el POS	4 (3)
	Valoraciones por especialistas	4 (3)
	Terapias físicas	2 (1,5)

IPS: institución prestadora de servicios de salud; POS: plan obligatorio de salud

A nivel social, la mayoría (98,5 %; 132/134) de los participantes refirieron no tener impedimentos familiares o religiosos para recibir el tratamiento de la enfermedad. Solo 3 de cada 10 pacientes han recibido información sobre los derechos de las personas con enfermedades huérfanas o raras de los diferentes medios de comunicación, y una minoría (10,4 %; 10/134) pertenece a algún grupo o fundación que fomenta la garantía de derechos a la población con este tipo de enfermedades.

En cuanto a los aspectos relacionados con el conocimiento y la cultura, el 38 % (51/134) de los participantes desconoce la definición de enfermedades huérfanas o raras, el 15,7 % (21/134) no recibió información sobre el diagnóstico y tratamiento a seguir por parte del profesional de la salud y al 25,4 % (34/134) no le explicaron las diferentes opciones terapéuticas.

Con relación a las barreras percibidas en la prestación de los servicios de salud, el 38,8 % (52/134) refiere que se ha tardado un mes o más tiempo entre la solicitud de un procedimiento por parte del médico especialista hasta lograr la autorización por parte de la aseguradora; asimismo, el 45,5 % (61/134) refiere que les ha tomado más de un mes entre la autorización de la aseguradora y la consulta con el médico especialista, destacándose el 15 % con tiempos superiores a 4 meses.

El 25,4 % (34/134) de los pacientes anotó que no les entregaron los medicamentos por parte de la aseguradora por la falta de disponibilidad o de inclusión en el plan de beneficios, entre otras limitantes. Aproximadamente, la mitad (47 %; 63/134) de los pacientes manifestó haber presentado problemas o dificultades después de la consulta con el médico especialista, encabezadas por los trámites excesivos, la falta de autorizaciones y la dificultad en disponibilidad de dinero para los copagos.

### Según el área de residencia

De los 134 participantes, el 38,1 % (51/134) vive en el área rural y, comparados con las personas que viven en el área urbana, tienen mayores probabilidades de estar afiliados al régimen subsidiado (OR = 9,92; IC_95%:_ 3,1440,7), de padecer discapacidad (OR = 4,12; IC_95%_: 1,09-22,9) y de presentar impacto negativo en el entorno familiar (OR = 2,88; IC_95%_: 1,25-6,88).

A su vez, las personas que viven en el área rural han presentado mayor porcentaje de hospitalizaciones que las del área urbana (39,2 %; 20/51) y el 28,9 % (24/83), respectivamente, y de ser beneficiarias de ayudas gubernamentales (27,5 %; 14/51) y el 13,2 % (11/83), respectivamente, sin diferencias estadísticamente significativas.

Las personas que viven en el área rural presentaron significativamente más probabilidades de experimentar barreras para acceder a los servicios de salud que los habitantes del área urbana, entre ellas:

1. el tiempo mayor a una hora para asistir a la institución prestadora del servicio de salud (OR = 2,34; IC_95%:_ 1,08-5,11);
2. el no contar con transporte propio (OR = 3,2; IC_95%_: 1,20-9,44);
3. la limitación en el acceso a vías hacia la institución prestadora del servicio de salud (OR = 10,76; IC_95%_: 4,36-27,01);
4. la escasez de dinero para el transporte (OR = 2,84; IC_95%:_ 1,26-6,52);
5. otros gastos económicos complementarios al tratamiento (OR = 3,05; IC_95%_: 1,39-6,74),
6. la falta de información por los medios de comunicación sobre los derechos de las personas con enfermedades huérfanas o raras (OR = 3,2; IC_95%_: 1,26-8,86),
7. la no pertenencia a una fundación de pacientes con enfermedades huérfanas o raras (OR = 9,28; IC_95%_: 1,29-402,3);
8. el desconocimiento de la definición de enfermedades huérfanas o raras (OR = 7,76; IC_95%_: 3,29-18,5),
9. la ausencia de información por parte del profesional de salud que no ha hablado de enfermedades huérfanas o raras (OR = 4,1; IC_95%_: 1,39-12,9) y
10. el desconocimiento de las opciones de tratamiento (OR = 6,48; IC_95%_: 2,55-17,07) (cuadro 4).

**Cuadro 4:** Barreras que enfrentan las personas con enfermedades huérfanas para acceder a los servicios de salud según la ubicación geográfica de la residencia (N = 134)

Variables	Rural	Urbano	OR	IC95 %	p
	(n = 51)	(n = 83)			
	n (%)	n (%)			
Régimen subsidiado	47 (92)	45 (54.2)	9,92	3,14 - 40,73	< 0,001
Hospitalizado	20 (39,2)	24 (28,9)	1,58	0,70 - 3,52	0,21
Presencia de discapacidad	48 (94,1)	66 (79,5)	4,12	1,09 - 22,96	0,021
Impacto en el entorno familiar	39 (76,5)	44 (53)	2,88	1,25 - 6,88	0,006
Ayuda gubernamental	14 (27,5)	11 (13,2)	2,47	0,93 - 6,64	0,04
Tiempo mayor de 1 hora para asistir a la IPS	31 (60,8)	33 (39,8)	2,34	1,08 - 5,11	0,018
No cuenta con transporte propio.	44 (86,3)	55 (66,3)	3,2	1,20 - 9,44	0,01
Limitación en el acceso a vías a la IPS	34 (66,6)	13 (15,7)	10,76	4,36 - 27,01	< 0,001
Escasez de dinero para el transporte	37 (72,5)	40 (48,2)	2,84	1,26 - 6,52	0,005
Otros gastos económicos	29 (56,9)	25 (30,1)	3,05	1,39 - 6,74	0,002
Falta de información en los medios de comunicación	43 (84,3)	52 (62,6)	3,2	1,26 - 8,86	0,007
No pertenencia a una fundación de pacientes.	50 (98)	70 (84,3)	9,28	1,29 - 402,3	0,011
Desconocía la definición de enfermedad huérfana.	34 (66,7)	17 (20,5)	7,76	3,29 - 18,5	< 0,001
El profesional de salud no ha hablado de enfermedad huérfana.	14 (27,4)	7 (8,4)	4,1	1,39 - 12,9	0,003
Desconocía las opciones de tratamiento.	24 (47,1)	10 (12)	6,48	2,55 - 17,07	< 0,001

IC: intervalo de confianza; IPS: institución prestadora de servicios de salud; OR: odds ratio

### Según el régimen de afiliación

En comparación con el grupo de personas afiliadas al régimen con capacidad de pago, el grupo de personas afiliadas al régimen subsidiado tuvo más probabilidades de presentar dificultades para contar con dinero para transportarse hacia la institución prestadora del servicio de salud (OR = 2,75; IC_95%_: 1,21-6,27), ausencia de información sobre la complejidad de la enfermedades huérfanas o raras por parte del médico (OR = 5,2; IC_95%_: 1,14-47,9), desconocimiento de las opciones de tratamiento (OR = 4,59; IC_95%_: 1,44-19,17), falta de entrega de los medicamentos (OR = 4,59; IC_95%_: 1,44-19,17) y otros problemas relacionados con la entrega o disponibilidad del tratamiento ordenado (OR = 3,66; IC_95%_: 1,54-9,03).

Las personas afiliadas al régimen de salud con capacidad de pago, en comparación con los afiliados al régimen subsidiado, tuvieron mayores probabilidades de presentar barreras para acceder a los servicios de salud, lo cual se hace evidente con la necesidad de interponer tutelas para lograr atención en salud (OR = 3,08 ;IC_95%_: 1,27-7,38), pagos de cuotas moderadoras y copagos (OR = 6,98 ; IC_95%_: 2,85-17,18), pago de medicamentos o procedimientos no autorizados por la aseguradora (OR = 2,91; IC_95%_: 1,27-6,64), tiempo mayor de una semana para lograr la autorización de un procedimiento o medio de diagnóstico por parte de la aseguradora (OR = 2,85; IC_95%_: 1,12-7,91) y tardar más de un mes entre la autorización por la aseguradora y la valoración por el médico especialista (OR = 2,64; IC_95%_: 1,17-6,04).

Aunque no existe una diferencia estadísticamente significativa, los afiliados del régimen con capacidad de pago requirieron trasladarse a otro departamento para recibir tratamiento para su enfermedad, evidenciando 5 puntos porcentuales mayor que el régimen subsidiado (cuadro 5).

**Cuadro 5:** Barreras que enfrentan las personas con enfermedades huérfanas para acceder a los servicios de salud según el régimen de salud (N = 134)

Variables	Régimen de salud		OR	IC	95 %	p
	Subsidiado Capacidad de pago					
	(n = 92)	(n = 42)				
	n (%)	n (%)				
Escasez de dinero para el transporte	60 (65,2)	17 (40,5)	2,75	1,21 -	6,27	0,007
El profesional de salud no ha hablado de enfermedades huérfanas.	19 (20,6)	2 (4,8)	5,2	1,14 -	47,9	0,018
Desconoce opciones de tratamiento.	30 (32,6)	4 (9,5)	4,59	1,44 -	19,17	0,004
Falta de entrega de los medicamentos	30 (32,6)	4 (9,5)	4,59	1,44 -	19,17	0,004
Problemas con el tratamiento	52 (56,5)	11 (26,2)	3,66	1,54 -	9,03	0,001

IC: intervalo de confianza; OR: *odds ratio*

## Discusión

Las enfermedades huérfanas o raras son un grupo de enfermedades que, por sus características particulares, representan desafíos sociales y de salud muy distintas a las de las enfermedades comunes ^1^^-^^3^. Los participantes del presente estudio se encuentran ubicados en los cuatro primeros cursos de vida, presentan diversidad de diagnósticos y reportan haber requerido atención médica especializada. Más del 80 % (114/134) de los participantes presentaron algún tipo de discapacidad. Sumado a esto, los pacientes debieron enfrentar barreras de tipo geográfico y económico para acceder a los servicios de salud, siendo estas más acentuadas en los residentes del área rural y en los afiliados al régimen subsidiado.

Por consiguiente, la presente investigación era necesaria y pertinente, dado que las personas con enfermedades huérfanas o raras son consideradas sujetos de protección especial por parte del Estado colombiano como lo establece la Ley 1751 de 2015 ^10^.

En Colombia, la Resolución 023 de 2023 reconoce 2247 enfermedades huérfanas o raras de especial interés para el sistema de salud ^6^; no obstante, ninguna de estas enfermedades cuenta con una ruta de atención justificada en las particularidades heterogéneas que este grupo de enfermedades presentan, y solo se cuenta con el plan nacional de gestión para las enfermedades huérfanas o raras, cuyo objeto es formular acciones que promuevan el acceso efectivo a la atención integral de salud de las personas con alguna de estas enfermedades ^1^.

A nivel clínico, la especialidad médica tratante de mayor prevalencia en los participantes fue genética, seguida de neurología, lo que coincide con lo reportado en el Registro Mexicano de Pacientes con Enfermedades Raras ^19^, dado que muchas de ellas tienen un componente hereditario, que requiere de pruebas genéticas para el diagnóstico y determinación del tratamiento. Hubo predominio (91,8 %; 123/134) fue de pacientes de los estratos socioeconómicos más bajos (I y II), lo que les atribuye un carácter de vulnerabilidad socioeconómica, contrario a los pacientes del registro mexicano ^19^.

Ahora bien, son diversos los estudios del ámbito nacional e internacional que han evidenciado las barreras que se imponen en la atención en salud. En nuestro estudio, se evidenció que los pacientes que residen en el área rural tienen mayores probabilidades de experimentar barreras de acceso de tipo geográfico y organizativo, lo que les implica una mayor inversión de tiempo y de dinero.

En Colombia, la red hospitalaria de mayor complejidad y los médicos especialistas se concentran en las ciudades capitales, y las personas de zonas rurales generalmente carecen de los medios económicos y logísticos para ser atendidos en la ciudad. Esto concuerda con otros estudios del contexto nacional e internacional, que indicaron que las largas distancias por recorrer, sumadas al costo financiero personal, constituyeron las principales barreras para acceder a los médicos especialistas ^20^^-^^26^.

Por otro lado, el 92,2 % (47/51) de quienes residen en el área rural pertenece al régimen subsidiado, similar a un estudio realizado en Australia en el que las familias en áreas regionales y remotas tenían significativamente menos probabilidades de tener un seguro médico privado y un acceso adecuado a los servicios de salud, y mayores probabilidades de informar tres o más barreras de acceso a la atención médica que las familias que viven en ciudades principales ^25^.

En nuestro estudio, más de la mitad (56,5 %; 52/92) de quienes pertenecen al régimen subsidiado reportaron tener problemas con el tratamiento, lo que se relaciona con los excesivos trámites administrativos en las autorizaciones de las tecnologías no incluidas en el plan de beneficios; mientras que el 32,6 % (30/92) reportó problemas relacionados con la falta de entrega de los medicamentos. Estas diferencias muestran que, si bien desde el 2012 se unificó el plan de beneficios en salud del régimen subsidiado con el del régimen contributivo ^27^, las barreras administrativas, la negación de la prestación de servicios y las barreras económicas siguen siendo mayores para los afiliados al régimen subsidiado ^22^^,^^23^, lo que impacta la oportunidad de acceder a los tratamientos necesarios para este tipo de enfermedades, llevándolos a incurrir en gastos adicionales o a abandonar sus procesos.

El acceso a los medicamentos es un problema trascendental en varios sistemas de salud, e incide en el control de las enfermedades y en la prevención de complicaciones. Esto ocurre con las enfermedades en general, pero causa mayor preocupación en las enfermedades huérfanas o raras, debido a los altos costos y a la escasa disponibilidad de los medicamentos, además de los trámites administrativos, lo que concuerda con las barreras para acceder a los medicamentos reportadas en un país desarrollado ^25^.

En Colombia, el principio de accesibilidad a los servicios de salud está normado en el Decreto 1652 de 2022, el cual exceptúa del cobro de cuotas moderadoras y copagos a las personas con enfermedades huérfanas o raras afiliadas al SGSSS ^28^, con el fin de evitar barreras en la prestación del servicio de salud como las que evidencian los presentes resultados. Por consiguiente, las fallas en la aplicación y cumplimiento de la normatividad en mención se manifiestan como una barrera para la garantía del derecho a la salud y puede agudizar la complejidad del proceso que deben llevar los pacientes y sus familias.

Las barreras de tipo económico demostraron que el 57,5 % (77/134) manifestaron escasez de dinero para transportarse hacia los servicios de salud. Asimismo, el 32,8 % (44/134) de los pacientes ha tenido que asumir los gastos de viáticos para recibir el tratamiento, lo cual es similar a lo reportado en un estudio de España ^26^ y en el estudio ENSERio (estudio sobre la situación de necesidades sociosanitarias de las personas con enfermedades raras), realizado en Colombia ^29^, en el que el mayor porcentaje de costos asumidos por los pacientes corresponde al transporte, el cual no se encuentran dentro del plan de beneficios. Estos gastos adicionales repercuten en la continuidad y sostenibilidad del tratamiento.

Pese a los gastos de bolsillo que imponen las barreras de acceso (40,3 %; 54/134) y a las características de vulnerabilidad socioeconómica -por estrato- (91,8 %; 123/134) de los participantes, se reportó un impacto positivo significativo en las relaciones intrafamiliares y en aspectos emocionales, similar a lo arrojado en un estudio en España 30. Estos datos evidencian que la atención en salud de estos pacientes por parte de un equipo multidisciplinario debe contemplar e intervenir de forma profunda en los aspectos emocionales y sociales del paciente y de su familia ^2^^,^^4^^,^^29^.

La presencia de discapacidad en el 85,1 % (114/134) de los participantes agudiza las necesidades de esta población por la limitada posibilidad de trabajar y generar recursos, sumado a la necesidad de algunos pacientes de tener un cuidador permanente, afectando aún más la economía y el estado emocional familiar. Además, existe falta de apoyo gubernamental en la asignación de subsidios que ayuden a disminuir la carga de la enfermedad a nivel individual y familiar ^26^. En Australia, por el contrario, la gran mayoría de pacientes (79 %) con diagnósticos similares reciben apoyo financiero de programas gubernamentales ^25^.

El pertenecer a organizaciones de pacientes contribuye a reducir el vacío de conocimiento básico de la enfermedad y a velar por el reconocimiento de los derechos de los pacientes ante la ignorancia en el tema de las enfermedades huérfanas o raras, incluso entre los mismos profesionales de la salud ^24^^,^^26^^,^^31^^,^^32^. Sin embargo, solo el 10,4 % de los pacientes con este tipo de enfermedades pertenece a algún grupo social o fundación, lo que evidencia una baja participación en los grupos de apoyo de pares. Esto podría atribuirse al estigma social o al desconocimiento o inexistencia de estas comunidades con interés común ^24^^,^^26^.

Por ello, la capacitación sobre enfermedades huérfanas o raras a estudiantes y profesionales de la salud, especialmente a quienes trabajan en atención primaria ^24^^,^^33^, resulta fundamental, sobre todo en el área rural donde la falta de información para pacientes y familias es mayor.

Por otra parte, en Colombia se ha reconocido el derecho fundamental a la salud mediante la Ley Estatutaria 1751 de 2015, que permite que los ciudadanos interpongan recursos legales cuando les vulneran la atención en salud ^10^^,^^34^. En el presente estudio, el 26,9 % (36/134) de los participantes se vieron obligados a recurrir a tutelas; de estos, el 69,4 % (25/36) corresponde a personas del área urbana y el 44,4 % (16/36) del régimen de capacidad de pago, lo que puede atribuirse a mayor conocimiento circulante sobre esta herramienta jurídica y mayor accesibilidad a procesos jurídicos en la ciudad. Esto va en línea con la afirmación de que en el área urbana hay mayor población con capacidad de pago, y, por tanto, más facilidad para acceder a estos trámites legales; mientras que en la ruralidad predomina el régimen subsidiado, con mayores barreras geográficas que impiden su uso ^23^.

Según la Ley Estatutaria 1618 del 2013 ^34^ y la Resolución 1197 de 2024 ^35^, a las personas con discapacidad se les debe garantizar el derecho a obtener el certificado de discapacidad que, a su vez, permite mantener actualizada la información de la plataforma de registro de la localización y caracterización de las personas con discapacidad.

En el presente estudio, el 74,6 % (100/134) de los pacientes requiere el certificado de discapacidad, pero no cuentan con este documento debido, principalmente, a la falta de orientación para gestionarlo. Por tanto, resulta urgente fortalecer el talento humano en salud sobre el procedimiento de la generación de la certificación y la actualización de datos en la plataforma de registro de la localización y caracterización de las personas con discapacidad. Esta plataforma representa una fuente de información para la formulación, implementación y seguimiento de políticas públicas, planes, programas y proyectos ^24^ orientados a la garantía de los derechos de las personas con discapacidad según la Resolución 1239 de 2022 ^36^; además, sirve como medio de verificación de la existencia de discapacidad o para la priorización de programas sociales.

Las fortalezas del presente estudio incluyen el abordaje integral para analizar las barreras que enfrentan las personas con enfermedades raras, al considerar múltiples dimensiones como las características sociodemográficas, el tipo de afiliación, las redes de apoyo, las dificultades de transporte, los procesos de autorización y el acceso a especialistas. No se identificaron investigaciones previas en el contexto local con este enfoque amplio, lo que resalta su valor para orientar políticas y mejorar la atención a esta población.

Las limitaciones fueron la falta de aleatorización para seleccionar la muestra, dado que se tomaron datos disponibles de personas con enfermedades huérfanas o raras que tenían información completa en la base de datos y no fue posible analizar los resultados por subgrupos de enfermedades.

Aunque los problemas de las enfermedades huérfanas o raras suelen ser compartidos por la mayoría de los afectados, y a pesar de que el objetivo de este estudio no fue establecer diferencias entre manifestaciones clínicas, se considera que el análisis global realizado permitió conocer mejor la realidad de los afectados.

En conclusión, las personas con enfermedades huérfanas o raras en el departamento del Huila enfrentan una acentuada vulnerabilidad, determinada por factores sociodemográficos y por las características clínico-terapéuticas propias de este tipo de enfermedades.

A pesar de que la normatividad colombiana reconoce y prioriza la atención para esta población de pacientes, persisten barreras significativas para el acceso oportuno al diagnóstico y el tratamiento. Esta situación se agrava en los contextos rurales y en la población afiliada al régimen subsidiado, lo cual repercute negativamente en la adhesión terapéutica, incrementa el riesgo de complicaciones y favorece la progresión de la enfermedad.

Para lograr diagnósticos oportunos de las enfermedades huérfanas o raras, atención de calidad y empatía hacia el paciente, es indispensable que la atención en salud esté orientada a cubrir las necesidades de los pacientes y de sus familias, así como optimizar la toma de decisiones en el marco de política pública, el fomento de la investigación y el desarrollo de capacidades del talento humano en salud desde el pregrado universitario.

## References

[1] Ministerio de Salud y Protección Social (2024). Plan nacional de gestión para las enfermedades huérfanas/raras.

[2] Castañeda C, Holguín A, Roselli D (2019). Enfermedades raras: del diagnóstico a las políticas públicas. Primera edición.

[3] Mancera CL, Seguanes CC, Lerma Y (2021). Acceso a los servicios de salud de personas diagnosticadas con enfermedades huérfanas en Colombia. Rev CIES.

[4] Fundación Retorno Vital Proyecto de colaboración: ruta enfermedades huérfanas en Colombia.

[5] Instituto Nacional de Salud Informe de evento enfermedades huérfanas-raras: 2022.

[6] Ministerio de Salud y Protección Social Resolución 023 de 2023.

[7] Congreso de Colombia Ley 1392 de 2010.

[8] Instituto Nacional de Salud Protocolo de vigilancia en salud pública de enfermedades huérfanas-raras, versión 06.

[9] Departamento Administrativo de la Función Pública Decreto 2106 de 2019.

[10] Congreso de Colombia Ley estatutaria 1751 de 2015.

[11] Frenk J. (1985). El concepto y medición de la accesibilidad. Salud Publ Méx.

[12] Andersen RM (1995). Revisiting the behavioral model and access to medical care: Does it matter?. J Health Soc Behav.

[13] Graves A (2009). A model for assessment of potential geographical accessibility: A case for GIS. Online J Rural Nurs Health Care.

[14] Congreso de Colombia Ley 1581 de 2012.

[15] Ministerio de Salud y Protección Social Resolución 3280 de 2018.

[16] Superintendencia Nacional de Salud (2023). Glosario.

[17] Departamento Nacional de Planeación (2021). Análisis de la estructura familiar en Colombia a partir de registros administrativos y del programa *Mi familia* del ICBF. Observatorio de familias.

[18] Ministerio de Salud y Protección Social Resolución 8430 de 1993.

[19] Calvo CE, Gonzaga-Jauregui C (2024). First year results and insights from the Mexican Rare Disease Patient Registry. Rare.

[20] Best S, Vidic N, An K, Collins F, White SM (2022). A systematic review of geographical inequities for accessing clinical genomic and genetic services for non-cancer related rare disease. Eur J Hum Genet.

[21] Echevarría AF, Lopera DA (2022). Barreras de acceso a los servicios de salud que enfrentan las personas que padecen enfermedades huérfanas en Colombia.

[22] Bran L, Valencia A, Palacios L, Gómez S, Acevedo Y, Arias C (2020). Barreras de acceso del sistema de salud colombiano en zonas rurales: percepciones de usuarios del régimen subsidiado. Revista Hacia la Promoción de Salud.

[23] Departamento Administrativo Nacional de Estadística (2023). Encuesta Nacional de Calidad de Vida (ECV).

[24] Tsitsani P, Katsaras G, Soteriades ES (2023). Barriers to and facilitators of providing care for adolescents suffering from rare diseases: A mixed systematic review. Pediatr Rep.

[25] Teutsch S, Zurynski Y, Eslick GD, Deverell M, Christodoulou J, Leonard H (2023). Australian children living with rare diseases: Health service use and barriers to accessing care. World J Pediatr.

[26] Güeita-Rodriguez J, Famoso-Pérez P, Salom-Moreno J, Carrasco-Garrido P, Pérez-Corrales J, Palacios-Ceña D (2020). Challenges affecting access to health and social care resources and time management among parents of children with rett syndrome: A qualitative case study. Int J Environ Res Public Health.

[27] Comisión de Regulación en Salud Acuerdo 032 de 2012.

[28] Ministerio de Salud y Protección Social Decreto 1652 de 2022.

[29] Salazar LV, Quiroga M, Mesa M, Suárez F, Alianza Iberoamericana de Enfermedades Huérfanas (ALIBER) Estudio ENSERio LATAM: informe capítulo Colombia.

[30] Giménez-Lozano C, Páramo-Rodríguez L, Cavero-Carbonell C, Corpas-Burgos F, López- Maside A, Guardiola-Vilarroig S (2022). Rare diseases: Needs and impact for patients and families: A cross-sectional study in the Valencian region, Spain. Int J Environ Res Public Health.

[31] Gittus M, Chong J, Sutton A, Ong ACM, Fotheringham J (2023). Barriers and facilitators to the implementation of guidelines in rare diseases: A systematic review. Orphanet J Rare Dis.

[32] Lopes MT, Koch VH, Sarrubbi-Junior V, Gallo PR, Carneiro-Sampaio M (2018). Difficulties in the diagnosis and treatment of rare diseases according to the perceptions of patients, relatives and health care professionals. Clinics.

[33] Wainstock D, Katz A (2023). Advancing rare disease policy in Latin America: A call to action. Lancet Reg Health Am.

[34] Congreso de Colombia Ley estatutaria 1618 de 2013.

[35] Ministerio de Salud y Protección Social Resolución 1197 de 2024.

[36] Ministerio de Salud y Protección Social Resolución 1239 de 2022.

